# Human Population Density and Extinction Risk in the World's Carnivores

**DOI:** 10.1371/journal.pbio.0020197

**Published:** 2004-07-13

**Authors:** Marcel Cardillo, Andy Purvis, Wes Sechrest, John L Gittleman, Jon Bielby, Georgina M Mace

**Affiliations:** **1**Department of Biological Sciences, Imperial College LondonAscot, United Kingdom; **2**Institute of Zoology, Zoological Society of LondonLondon, United Kingdom; **3**IUCN Global Mammal Assessment, Species Survival Commission of IUCN and Conservation International Center for Applied Biodiversity Science Biodiversity Assessment Unit, Center for Applied Biodiversity, Conservation InternationalWashington, District of Columbia, United States of America; **4**Department of Biology, University of VirginiaCharlottesville, VirginiaUnited States of America

## Abstract

Understanding why some species are at high risk of extinction, while others remain relatively safe, is central to the development of a predictive conservation science. Recent studies have shown that a species' extinction risk may be determined by two types of factors: intrinsic biological traits and exposure to external anthropogenic threats. However, little is known about the relative and interacting effects of intrinsic and external variables on extinction risk. Using phylogenetic comparative methods, we show that extinction risk in the mammal order Carnivora is predicted more strongly by biology than exposure to high-density human populations. However, biology interacts with human population density to determine extinction risk: biological traits explain 80% of variation in risk for carnivore species with high levels of exposure to human populations, compared to 45% for carnivores generally. The results suggest that biology will become a more critical determinant of risk as human populations expand. We demonstrate how a model predicting extinction risk from biology can be combined with projected human population density to identify species likely to move most rapidly towards extinction by the year 2030. African viverrid species are particularly likely to become threatened, even though most are currently considered relatively safe. We suggest that a preemptive approach to species conservation is needed to identify and protect species that may not be threatened at present but may become so in the near future.

## Abstract 2

As human populations increase, the importance of intrinsic biological factors in determining which carnivores become extinct will also rise - an interaction that makes some species more threatened than previously thought

## Introduction

Mammals have been severely affected by the current extinction crisis: around a quarter of extant species are considered to be threatened with extinction (Hilton-Taylor 2000). Understanding the ecological processes that cause some species to decline, while others remain relatively safe, may help to predict future declines and focus conservation efforts on species in urgent need. The underlying cause of virtually all recent and ongoing declines of mammal species is the growth of human populations and associated impacts such as habitat loss, hunting, and the spread of invasive species. Threatening processes such as these vary in intensity across the surface of the Earth, and species that inhabit more heavily impacted regions are expected to have a higher risk of extinction (Forester and Machlis 1996; Woodroffe 2000; Brashares et al. 2001; Harcourt et al. 2001; McKinney 2001; Ceballos and Ehrlich 2002; Harcourt and Parks 2002; Parks and Harcourt 2002).

Although exposure to threatening processes is the ultimate cause of extinction, a species' biology determines how well it is able to withstand the threats to which it is exposed. Biological traits that confer ecological flexibility and allow populations to recover rapidly from depletion may offer a degree of protection from external threats. A number of recent studies have linked variation in extinction risk or decline among species to biological traits (Gaston and Blackburn 1995; Bennett and Owens 1997; Owens and Bennett 2000; Purvis et al. 2000; Cardillo and Bromham 2001; Cardillo 2003; Fisher et al. 2003; Jones et al. 2003), and, indeed, biology accounts for over a third of the variation in extinction risk among carnivore and primate species (Purvis et al. 2000). However, only one study to date, using Australian marsupials (Fisher et al. 2003), has explicitly examined the relative importance of biological versus external, anthropogenic predictors of extinction risk. Hence, we know little about the extent to which adding external predictors might increase the explanatory power of models of extinction risk based on biology alone. Furthermore, we do not know whether the combined effects of biological and external predictors are simply additive, or whether interactions exist: does the influence of biology vary depending on the degree of external threat a species faces?

Here we present a global-scale analysis of biological and external predictors of extinction risk in the mammal order Carnivora. As well as including many symbols of conservation such as the giant panda, tiger, and sea otter, carnivores in general are a good model taxon for the development of a predictive science of conservation: their biology and phylogeny are well studied, they are near-global in distribution, they represent a wide range of biological strategies, and they include species at all levels of extinction risk. Our analysis emphasizes those threatened species that have suffered measurable declines, rather than those simply with small populations or ranges that may be considered “naturally” rare. We use human population density (HPD) as a summary measure of anthropogenic impact. Although not all types of impact are necessarily associated with high-density human populations, on a global scale HPD is more reliably quantified than direct threatening processes such as habitat loss or hunting, which are difficult to measure accurately in ways that are consistent across regions and biomes. Therefore, HPD represents one of the best available means of summarizing the impact faced by mammal species on a global scale. At local or regional scales, high HPD is often associated with some measure of mammal decline (Forester and Machlis 1996; Woodroffe 2000; Brashares et al. 2001; Harcourt et al. 2001; McKinney 2001; Ceballos and Ehrlich 2002; Harcourt and Parks 2002; Parks and Harcourt 2002). Here we ask whether HPD influences carnivore extinction risk at the species level, whether it is more or less important than species biology, and how biology interacts with HPD to determine risk.

## Results

We followed the international standard for species-level extinction risk classification, the IUCN Red List (Hilton-Taylor 2000), which has also been used in previous studies of species-level extinction risk (Purvis et al. 2000; Harcourt and Parks 2002; Jones et al. 2003). We used multiple linear regression to find minimum adequate models (MAMs) predicting extinction risk from HPD and a set of biological traits. Confounding effects of phylogeny were controlled for by calculating phylogenetically independent contrasts in all variables before analysis. Using a Geographic Information System, we derived seven summary measures of HPD for each species: mean HPD across the species' geographic range and the proportion of the range with HPD of at least 2, 5, 10, 20, 50, and 100 people/km^2^. With the exception of the last two of these, all showed significant nonlinear effects on extinction risk as separate predictors (Table 1). In the multiple regression, however, biological variables were of overriding importance compared to HPD as predictors of extinction risk (Table 2). A MAM based on main effects alone explained 45.1% of variation in risk, with four biological variables independently associated with high extinction risk: small geographic range size, long gestation, low species population density and high trophic level. No HPD variables added significant explanatory power to this model. However, when interactions between HPD and biological variables were added to the model, a HPD–gestation length interaction was significant, and the variance explained by the model increased to 51.4% (Table 2). Using the Akaike information criterion (AIC), the model with the interaction term provided a better fit to the data (AIC = 57.38) than the model based on main effects only (AIC = 61.98). However, the partial variance explained by HPD (0.5%) in this model was very small compared to that explained by the combined biological variables (44%). So, although HPD variables were significant separate predictors of extinction risk, the independent effect of HPD virtually disappeared once the effects of biology were accounted for.

**Table 1:**
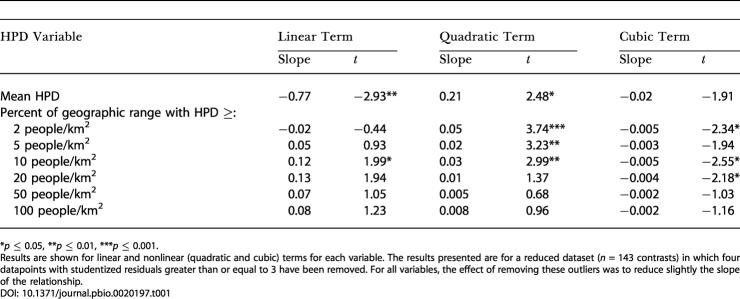
Regressions of Extinction Risk against HPD Using Phylogenetically Independent Contrasts

*p ≤ 0.05, **p ≤ 0.01, ***p ≤ 0.001

Results are shown for linear and nonlinear (quadratic and cubic) terms for each variable. The results presented are for a reduced dataset (*n* = 143 contrasts) in which four datapoints with studentized residuals greater than or equal to 3 have been removed. For all variables, the effect of removing these outliers was to reduce slightly the slope of the relationship

**Table 2:**
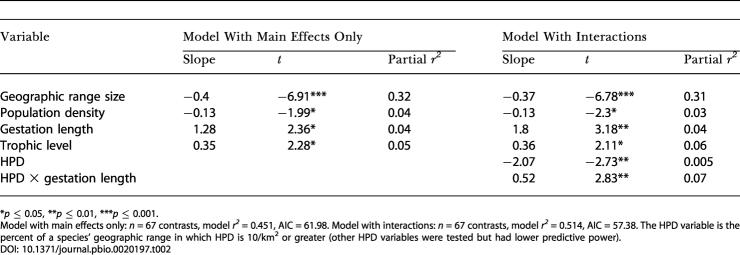
MAMs from Multiple Regression of HPD and Biological Predictors of Extinction Risk in Carnivores

*p ≤ 0.05, **p ≤ 0.01, ***p ≤ 0.001

Model with main effects only: *n* = 67 contrasts, model *r^2^* = 0.451, AIC = 61.98. Model with interactions: *n* = 67 contrasts, model *r^2^* = 0.514, AIC = 57.38. The HPD variable is the percent of a species' geographic range in which HPD is 10/km^2^ or greater (other HPD variables were tested but had lower predictive power)

The importance of interactions between HPD and biology was confirmed by separate analyses of the subsets of carnivore species with relatively low and high exposure to human populations (Table 3). For “low-exposure” species the final MAM included only two predictors, species population density and geographic range size, and explained 37.9% of the variation in extinction risk. However, for “high-exposure” species the model included geographic range size, species population density, and gestation length, and the explanatory power increased sharply, to 80.1%. Therefore, despite the fact that independent main effects of HPD were relatively unimportant, HPD did appear to be a significant modifier of the effects of biology on extinction risk.

**Table 3:**
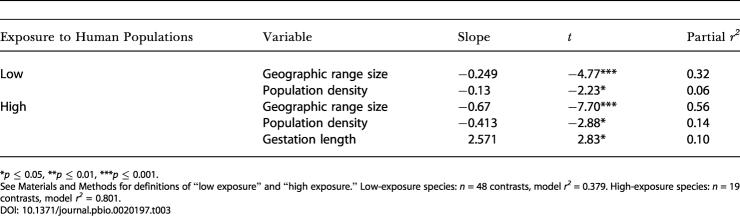
MAMs for Carnivore Species with Low and High Exposure to Human Populations

*p ≤ 0.05, **p ≤ 0.01, ***p ≤ 0.001

See Materials and Methods for definitions of “low exposure” and “high exposure.” Low-exposure species: *n* = 48 contrasts, model *r^2^* = 0.379. High-exposure species: *n* = 19 contrasts, model *r^2^* = 0.801

## Discussion

While the ultimate sources of current threats to species are virtually all anthropogenic, intrinsic ecological and life-history traits determine how well populations are able to withstand exposure to threatening processes. In our models, four biological traits accounted for nearly half of the variation in extinction risk among carnivore species. Small geographic ranges and low population densities determine the maximum population size a species can attain; gestation length is an important indicator of life-history speed (Gittleman 1993), which determines how quickly populations can recover from low levels; and extinction risk for species at high trophic levels may be compounded by their need for large hunting areas and their dependence on prey species that may themselves be threatened (Carbone and Gittleman 2002). Of these traits, geographic range size is of particular importance. As an example, the Ethiopian wolf *(Canis simensis)* is the most threatened species of the Canidae family despite the fact that its population density is not especially low, nor its gestation especially long, compared to other *Canis* species. However, it has a geographic distribution only a fraction of the size of its congenerics, and within that distribution is limited to afroalpine habitat (Sillero-Zubiri and Macdonald 1997).

In contrast, independent, main effects of HPD on extinction risk were weak once the effects of biology were accounted for: HPD explained only 0.5% of variation in extinction risk in the final model, compared to 44% for biological traits. That extinction risk in carnivores should be so strongly determined by biology rather than HPD is surprising, given that carnivores inhabit regions as disparate in human impact as western Europe and the Canadian Arctic, and that their requirements often conflict with human interests (Gittleman et al. 2001). Based on previous studies, HPD is expected to be a good proxy for threats to mammal populations (Forester and Machlis 1996; Woodroffe 2000; Brashares et al. 2001; Harcourt et al. 2001; McKinney 2001; Ceballos and Ehrlich 2002; Harcourt and Parks 2002; Parks and Harcourt 2002), and, indeed, it has been suggested that HPD be incorporated into a scheme for quantifying extinction risk for primate species (Harcourt and Parks 2002). However, no previous study has examined the effects of HPD on extinction risk after controlling for the effects of a wide range of biological traits. The strength of HPD as a predictor of risk may also be compromised by the fact that the relationship between HPD and threat intensity may be a complex one (McKinney 2001): for example, habitat loss, the most important threat to mammals (Hilton-Taylor 2000), is often not associated with high HPD. Recent work suggests that number of households may be a better demographic indicator of threat intensity than number of people (Liu et al. 2003), although this is based on coarse-scale, country-level data that cannot easily be incorporated into phylogenetically explicit analyses. Furthermore, differences in the degree of technological development of different human societies may contribute to differences in the effect of HPD. For example, a small human population with access to highly mechanized means of habitat destruction may have a level of impact on carnivores equal to that of a far larger or denser population without such means. Another possibility is that a species' current extinction risk status may reflect patterns of human impact in the past more closely than it does current impact. Extinction filter effects (Balmford 1996) may mean that the most vulnerable species have already disappeared or contracted away from regions of highest HPD, obscuring any underlying positive association between HPD and extinction risk. Unfortunately, the difficulty of reliably reconstructing historical ranges of species from available evidence probably precludes thorough testing of this idea.

Clearly, the importance of HPD on current extinction risk of carnivores is less as an independent main effect than as a modifier of the effects of biology. For “low-exposure” species (for which HPD was 10 people/km^2^ or higher in less than half of the range), life history did not predict extinction risk, and the only variables included in the model were those that determine the maximum population size a species can achieve (geographic range size and species population density). For “high-exposure” species, life history (gestation length) became additionally significant, and the explanatory power of the model as a whole was very high (80%). Evidently, a small species population contributes to high extinction risk no matter what the threat level, but where exposure to human populations is high, the disadvantage of a small species population is compounded by the disadvantage of a slow life history. This might suggest a difference in the major threat types experienced by carnivore species with different levels of exposure to human populations. For example, habitat loss, the predominant threat type for the great majority of mammals (Hilton-Taylor 2000), can be severe even far from centers of human population. Hence, species with restricted distributions and small populations may be susceptible to habitat loss even in regions of relatively low HPD. Where people are more numerous, species may be threatened by direct persecution and exploitation as well as habitat loss. In such regions, species with slow life histories and low population growth rates would become additionally susceptible. This could explain our finding that biology becomes a more powerful predictor of risk status as exposure to human populations increases.

If species in regions of high HPD are threatened by direct persecution and exploitation as well as habitat loss, we should expect top-level predators to be particularly threatened in these regions (Woodroffe 2000). Why, then, was trophic level not a predictor of extinction risk for “high-exposure” species? One possibility is an extinction filter effect, whereby species at the highest trophic levels, which live at low densities and are relatively rare, have already disappeared from regions of high HPD (Diamond 1984; Woodroffe 2001). This explanation appears to be supported by a negative correlation across species between trophic level and the proportion of a species' range with a HPD of 10 people/km^2^ or higher (r = −0.23, *p* = 0.003, d.f. = 166).

**Figure 1:**
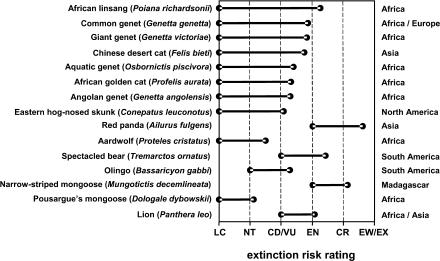
Carnivore Species Predicted to Move Most Rapidly towards Extinction by the Year 2030 Species listed are those expected to move from the “low-exposure” into the “high-exposure” group (see Materials and Methods for definitions), and for which the extinction risk rating is predicted to increase by at least one index value. Bars indicate the discrepancy between current Red List rating at the left, and the predicted rating at the right. General distributions of each species are shown on the far right. Abbreviations for Red List categories: LC, least concern; NT, near threatened; CD, conservation dependent; VU, vulnerable; EN, endangered; CR, critically endangered; EW, extinct in the wild; EX, extinct.

The strong effect of biological traits on extinction risk status for “high-exposure” species suggests that, as human populations increase globally over coming decades, the importance of biology in determining which species persist and which decline will also increase. This is worrying for species that possess traits making them more vulnerable to external threats, as their extinction risk can be expected to increase more sharply. Of particular concern are species that not only have unfavorable biology, but also live in regions of rapid human population growth. We have identified the carnivore species with the greatest expected increase in extinction risk over the next few decades, given HPD projected to the year 2030 based on recent growth rates. We acknowledge the problems with converting the ordinal categories of the Red List to an interval scale, and these have been discussed previously (Purvis et al. 2000). However, we emphasize that we are not attempting to make accurate quantitative predictions about the future status of species. We are simply illustrating a way of identifying those species likely to move most rapidly towards extinction in coming decades based on projected growth in human populations, all else being equal. Figure 1 shows those species with the greatest discrepancy between current and predicted risk status. These species are from a wide range of carnivore families, but the Viverridae (civets and genets) are particularly well represented: five of the top seven species on the list are viverrids. Most of the species in Figure 1 are from Africa, much of which has rates of human population growth far higher than the global average. It is particularly worrying that most are currently rated as “least concern” in the Red List, so they are unlikely to be receiving as much conservation attention as species currently rated as threatened. Furthermore, our estimates are conservative in that they treat species' geographic range sizes and population densities as static—they do not account for ongoing declines.

We conclude that there is no room for complacency about the security of species simply because they are not currently considered globally threatened. There is a strong case to be made for preemptive conservation of species, such as the African viverrids, that live in regions of rapid human population growth and have a biology predisposing them to decline. Preemptive action could include, for example, establishing population-monitoring programs, or listing species under national species protection laws on the basis of potential future susceptibility. Arguably, maintaining the stability of particularly susceptible species before they become threatened could be more cost-effective in the long term than postdecline attempts to rescue them from the brink of extinction.

## Materials and Methods

### 

#### Data

As the response variable in our analyses, we followed Purvis et al. (2000) in converting the IUCN Red List categories (Hilton-Taylor 2000) to a continuous linear index as follows: least concern = 0, near threatened = 1, conservation dependent/vulnerable = 2, endangered = 3, critically endangered = 4, extinct in the wild/extinct = 5. Among our predictor variables were geographic range size and species population density, both of which may contribute to the criteria for determining the Red List category (Hilton-Taylor 2000). To avoid potential circularity, our analyses excluded the 15% of carnivore species listed based on these criteria, and included threatened species only when they were listed under criterion A (a measured recent decline in geographic range or population size), which is independent of absolute geographic range or population density. We used the database of biological variables used by Purvis et al. (2000), updated to include more recently published information. This database consists of information compiled from the published literature on species' geographic range size, body size, interbirth interval, age at sexual maturity, litter size, gestation length, home range size, population density, group size, trophic level, activity timing, sociality, and island endemicity. Continuous variables were log-transformed before analysis.

For our measures of HPD, we used the Gridded Population of the World (CIESIN 2000), a spatially explicit global database of HPD for 1995, coarsened to a resolution of 0.5 ° × 0.5 ° to speed analyses. We used two methods to summarize the spatial variation in HPD within the geographic range of each species, each of which captures different aspects of HPD variation. Firstly, we used the log-transformed mean HPD across the geographic range of each species: this measure is sensitive to relatively small areas of very high HPD (e.g., around major cities). Secondly, we calculated the logit-transformed proportion of each species' range in which HPD exceeded a given threshold value. This measures a more explicitly spatial aspect of HPD variation and is less sensitive to small areas of very high HPD. Because it is difficult to know a priori the HPD threshold that is most critical to carnivore extinction risk, we repeated all analyses using threshold values of 2, 5, 10, 20, 50, and 100 people/km^2^. Geographic variation in both HPD and the distribution of threatened species may be confounded with net primary productivity (Balmford et al. 2001), so we included in the models a measure of actual evapotranspiration (AET) (UNEP 2003), as a proxy for primary productivity. The above calculations were all done with the Spatial Analyst extension in the program ArcGIS, using equal-area projections of the HPD map and each carnivore species' estimated current geographic distribution (compiled as part of the IUCN Global Mammal Assessment). The datasets used in the analyses are provided in Supporting Information (Datasets S1 and S2).

#### Analyses

To test the predictors of extinction risk we used linear regression through the origin (Garland et al. 1992) on phylogenetically independent contrasts generated using the program Comparative Analysis by Independent Contrasts (Purvis and Rambaut 1995). Although the extinction risk index itself does not evolve along phylogenies, it is closely associated with biological variables that do, making it necessary to use analyses that control for phylogeny to ensure statistical independence of data points (Jones et al. 2004; Purvis et al. 2000, 2004). The carnivore phylogeny of Bininda-Emonds et al. (1999) was used to define the contrasts, with branch lengths set to equal. The decision to use equal branch lengths was based on previous analyses (Purvis et al. 2000) using the same phylogeny and essentially the same biological dataset that showed that equal branch lengths gave contrasts with more homogeneous variances than those based on divergence times.

We first carried out univariate regressions of each HPD predictor against extinction risk (this had already been done for biological predictors by Purvis et al. [2000] using essentially the same dataset). We then combined external and biological predictors in multiple regressions. To find MAMs, we used backwards elimination of predictor variables from a full model (Crawley 2002)**.** The large number of missing values in the dataset, and the need to recalculate contrasts at each step, meant that this process could not be automated without discarding most of the information in the dataset. We therefore used the following manual procedure to find MAMs, following Purvis et al. (2000). We began by fitting a model with all predictors included, then identifying the predictor that contributed the smallest amount of marginal variance to the model. This predictor was then dropped, a new set of contrasts calculated, and the process repeated. In some cases dropping a predictor with many missing values resulted in a substantial increase in the number of contrasts at the next step; when this happened, other predictors previously dropped were reintroduced in turn and the model retested for each. A MAM was found when all remaining predictors contributed a significant (*p* ≤ 0.05) amount of variance to the model. Previously dropped predictors were then once again reintroduced in turn and the model retested each time. It should be noted that this method cannot guarantee to find the best-fitting model: it is essentially a heuristic search for the best model, and simulations on a dataset in which associations between variables are known would be needed to fully test the accuracy of the method.

To avoid potential problems of colinearity among the seven variables derived from HPD, the variables were included one at a time in the multiple regression models in the process of finding MAMs. At each step we substituted each of the seven HPD variables into the model in turn, retesting the model each time. Once the final MAM was found, we added terms describing the interactions between HPD and biological variables, again testing the model for significance each time.

Finally, we compared the predictive power of biological variables for subsets of species with low and high levels of exposure to human impact. “Low-exposure” and “high-exposure” species were defined, respectively, as species with less than or greater than 50% of their geographic range with HPD of at least 10 people/km^2^. The procedure for finding MAMs, using biological variables only, was then repeated for each of these two subsets of species.

#### Predictions of future risk increases

From global-scale spatial HPD data for 1990 and 1995 (CIESIN 2000) we calculated a mean annual rate of change, which we used to project HPD to the year 2030. For each species we then recalculated the proportion of the geographic range with HPD of at least 10 people/km^2^. Those species which moved from the “low-exposure” into the “high-exposure” group were identified, and the MAM for “high-exposure” species (Table 3) was used to predict extinction risk for these species. This method is more rigorous than simply identifying currently stable members of higher taxa that have declined in response to human population pressure, because it accounts for the unique biology and geographic distribution of each species.

## Supporting Information

Dataset S1 Definitions of Variable Names in the Dataset (1 KB TDS).

Dataset S2 External and Biological Data for Carnivores Used for Analyses (38 KB TDS).

## Abbreviations

HPD: human population density
MAM: minimum adequate model
AIC: Akaike information criterion
